# Plan robustness analysis for threshold determination of SGRT-based intrafraction motion control in 3DCRT breast cancer radiation therapy

**DOI:** 10.1186/s13014-023-02325-1

**Published:** 2023-09-22

**Authors:** Tim-Oliver Sauer, Wilhelm Stillkrieg, Oliver J. Ott, Rainer Fietkau, Christoph Bert

**Affiliations:** 1 Department of Radiation Oncology, Universitätsklinikum Erlangen, Friedrich-Alexander-Universität Erlangen-Nürnberg, Universitätsstraße 27, 91054 Erlangen, Germany; 2 Comprehensive Cancer Center Erlangen-EMN, Erlangen, Germany

**Keywords:** Treatment plan robustness, Surface guided radiation therapy, Intrafraction motion, Probabilistic dose evaluation

## Abstract

**Purpose:**

The goal of this study was to obtain maximum allowed shift deviations from planning position in six degrees of freedom (DOF), that can serve as threshold values in surface guided radiation therapy (SGRT) of breast cancer patients.

**Methods:**

The robustness of conformal treatment plans of 50 breast cancer patients against 6DOF shifts was investigated. For that, new dose distributions were calculated on shifted computed tomography scans and evaluated with respect to target volume and spinal cord dose. Maximum allowed shift values were identified by imposing dose constraints on the target volume dose coverage for 1DOF, and consecutively, for 6DOF shifts using an iterative approach and random sampling.

**Results:**

Substantial decreases in target dose coverage and increases of spinal cord dose were observed. Treatment plans showed highly differing robustness for different DOFs or treated area. The sensitivity was particularly high if clavicular lymph nodes were irradiated, for shifts in lateral, vertical, roll or yaw direction, and showed partly pronounced asymmetries. Threshold values showed similar properties with an absolute value range of 0.8 mm to 5 mm and 1.4° to 5°.

**Conclusion:**

The robustness analysis emphasized the necessity of taking differences between DOFs and asymmetrical sensitivities into account when evaluating the dosimetric impact of position deviations. It also highlighted the importance of rotational shifts, especially if clavicular lymph nodes were irradiated. A practical approach of determining 6DOF shift limits was introduced and a set of threshold values applicable for SGRT based patient motion control was identified.

**Supplementary Information:**

The online version contains supplementary material available at 10.1186/s13014-023-02325-1.

## Introduction

In radiation therapy, the treatment of cancer patients is preceded by an extensive preparation. The requirements of modern treatments demand a very precise calculation of the dose distribution and accurate and reproducible positioning of the patient for each treatment fraction. Image guidance methods like portal images or cone beam computed tomography (CBCT) have led to major improvements in patient positioning. At the same time, surface guided radiation therapy (SGRT) has become a powerful tool for patient positioning and control in the recent years [1–3]. In SGRT, the patient’s surface is captured optically, reconstructed and then registered to a reference surface, yielding correction shifts in six degrees of freedom (DOF) which correspond to the best match. These are used for prepositioning and can even substitute CBCT based positioning under certain circumstances [4, 5]. With its major advantages, i.e. real time acquisition and the absence of dose due to additional ionizing radiation exposure, it is predestined for continuous position control and even gating throughout the whole treatment process.

The general idea of patient position control is to keep deviations within certain bounds that assure appropriate delivery of the planned dose. In order to achieve that, it is necessary to access the robustness of a treatment plan, i.e. the resilience of the plan’s dose distribution to uncertainties [6]. Usually, deviations are accounted for by applying margins to the clinical target volume [7], although its geometric nature and the underlying dose cloud approximation have been criticized of not being able to describe deviations of the dose distribution to their full extent [6, 8, 9]. Concepts that go beyond this approach are usually probabilistic and involve the calculation of different scenarios [6, 8]. They are widely applied in proton therapy [10, 11] and increasingly in photon therapy, yet neither universal concepts nor safety requirements have been established [8], although many have been proposed [6].

Most of the few existent studies in the literature use scenario based (usually worst case) approaches in order to estimate the dosimetric effects of translational positioning uncertainties [9, 12–18], often in order to access and compare the robustness of treatment plans or to evaluate strategies of robust treatment planning [12, 13]. Hence the underlying question is if the treatment plan created with the treatment planning system (TPS) is robust enough in order to assure proper dose administration under inter- as well as intra-fractional positioning uncertainties encountered during therapy.

In this study, an inverse approach was followed, thus to access, originating from the robustness of a treatment plan, under which conditions SGRT based position control of the patient is accurate enough in order to assure correct dose delivery. The surface scanners usually allow for patient motion up to a default threshold value, without knowing in detail which effect these maximum shifts would have on the dose distribution. The robustness of clinical treatment plans, created for the treatment of breast cancer patients, were analyzed by simulating rigid motion in six DOF. Based on dose constraints, which had to be fulfilled after simulated patient motion, maximum shift threshold values were calculated. This yielded DOF specific limits that can serve as the upper bounds for SGRT based positioning (control). At the same time, the analysis can help identify plans that are less robust and may be selected for replanning.

## Materials and methods

**Fig. 1:**
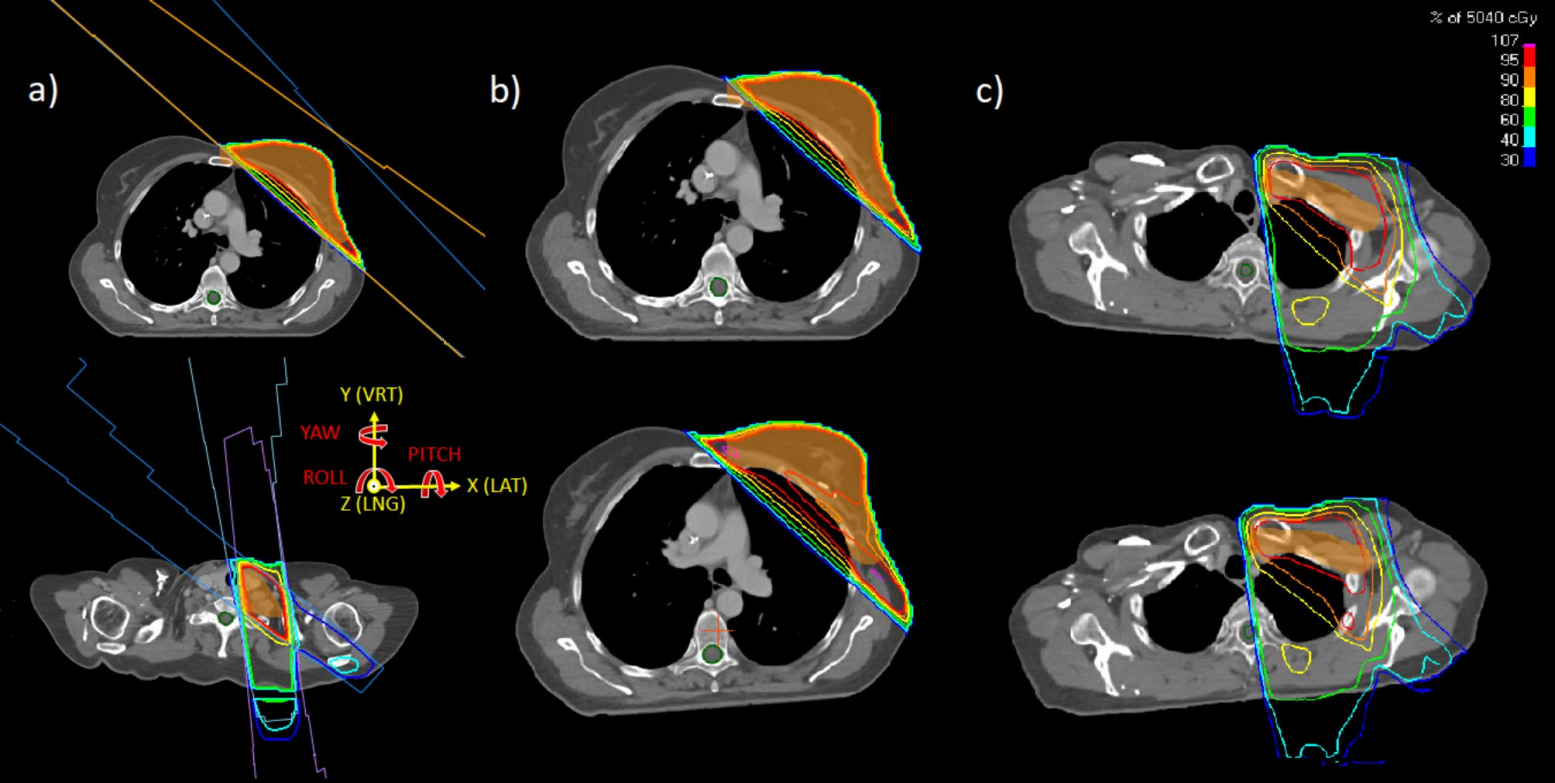
Illustration of the planning technique with beam outlines (**a**) of tangential fields (above) and additional fields for lymph node irradiation (below) and the coordinate system (middle); dose distribution of original treatment plan (**b**) in planning CT position (above) and for an exemplary 6-DOF shift (x,y,z = -3,-4,1 mm; yaw,pitch,roll = 1,-2,-4°) with obvious target volume coverage reductions (below), and for + 5° (**c**, above) and − 5° (**c**, below) rotational shift in roll around isocenter for illustration of the asymmetric effect on the spinal cord maximum dose

Data was obtained from treatments carried out as part of the clinical routine at the Department of Radiation Oncology, Universitätsklinikum Erlangen, on two Versa HD linacs (Elekta, Stockholm, Sweden), each equipped with the surface scanner AlignRT (Version 6.3.266; VisionRT, London, UK). Fifty female breast cancer patients, treated consecutively in the period of January to September 2021, have been included retrospectively in the study. There were 37/50 and 13/50 patients with left and right sided tumor location, 17/50 (all left-sided) and 33/50 with and without lymph node irradiation, respectively. There were no patients with right sided tumor location with lymph node irradiation in this cohort, because these patients were treated on a different linac. All patients were treated with 3D conformal beams, with two opposing main beams and additional beams for dose homogeneity. Patients that received lymph node irradiation were additionally treated with opposing beams at about 0° and 180°, and a lateral field (see Fig. 1a)). For the treatment in supine position with the arms placed above the head, a markerless workflow with indexed positioning devices (UNGER Medizintechnik, Mülheim-Kärlich, Germany) including a wingboard for reproducible arm positioning and SGRT-based positioning were used, as described in [19]. The region of interest determining the surface used for registration of the surface scanner comprised the breast and a rectangular area below the breast on both sides, as described in [20]. Depending on tumor stage, patients were treated either normo-fractionated (28 × 1.8 Gy) or hypo-fractionated (15 × 2.67 Gy). The patients were selected consecutively for this study, independently of their breast anatomy and dose prescription. Patients treated with VMAT were not included in this study because of the limited statistical significance due to their low number.

The dose calculations were performed with the clinical TPS RayStation (version 10B; RaySearch Laboratories, Stockholm). In order to evaluate the effect of rigid patient motion or misalignment, the original treatment beams were recalculated on the CT shifted by preset 6DOF shifts. For that, the planning CT was reimported a second time into the treatment plan and registered to the primary CT according to the preset shift, mimicking rigid patient motion. Recalculation of the beams on the shifted CT yielded a new dose distribution, which was evaluated as described below. An exemplary shift and the corresponding dose distribution are illustrated in Fig. 1a). A python script (CPython 3.6) was created, automatizing the above-mentioned steps for a range of shifts and all patients.

### 1DOF analysis

In a first step, only 1DOF at a time was varied in a range of (-5 mm, 5 mm) and (-5°, 5°) in 0.5 mm and 0.5° steps, respectively, keeping simulated patient shifts of all other DOF at zero. Rotations *R* were performed around treatment isocenter and in the order of yaw, pitch and roll, followed by translations *T*, resulting in a transformation matrix

$$M = T {T}_{iso} {R}_{roll} {R}_{pitch} {R}_{yaw} {T}_{-iso} ,$$

where *T*_*iso*_ and *T*_*− iso*_ are the isocenter shift and its inverse, respectively. It has been chosen in order to match the AlignRT system, which uses this order for its point cloud registration with an iterative closest point algorithm. Figure 1a) shows the coordinate system with indicated translations (x,y,z) and rotations (P,Y,R) = (pitch, yaw, roll).

For evaluation of the motion simulated dose distributions, the focus was put on target volume coverage C (V_95%_ of prescribed dose). It was evaluated for the volume of the PTV that was actually covered in the original treatment plan, resulting in a maximum PTV coverage equaling 1 for shifts of zero. Maximum shift (‘threshold’) values were identified on the basis of a pass rate concept prospectively established in coordination with senior physicians. The pass rate was defined as the proportion of shifts that were smaller or equal to the current maximum shift values and assured a relative target volume coverage of at least 95%. Threshold values were determined by the (absolute) maximum shift satisfying a pass rate of 95%, for positive and negative values of every DOF individually. Although it was not investigated in detail in this study, this concept can be adapted to organs at risk (OAR) dose constraints as well. For illustration, this had been done for 1DOF shifts for the spinal cord maximum dose, that was required to fulfil the clinical dose constraint of D_0.01 cm³_ ≤ 45 Gy. For details on other OARs, see the discussion section and additional files (2, 3, 4).

### 6DOF analysis

In a second step, we randomly sampled simultaneous 6DOF shifts in order to obtain representative values. We sampled 1000 shifts per patient from a uniform random distribution in the same range as the above mentioned 1DOF shifts. For every set of shifts, the target volume coverage was calculated. In the following, we applied an iterative algorithm to the accumulated data of all patients (or subgroups, respectively), in order to obtain a set of threshold values that fulfill the same 95% pass rate criterium as for the 1DOF analysis. For a certain set of threshold values

$${\overrightarrow{X}}_{6DOF}={({x}_{\pm }=({x}_{+},{x}_{-}),{y}_{\pm },{z}_{\pm },{yaw}_{\pm },{pitch}_{\pm },{roll}_{\pm })_{6DOF},}$$

the pass rate r of all randomly sampled shifts $\overrightarrow{S}$ that entered in the thereby defined interval, thus those satisfying ${{X}_{i-,6DOF}\le S}_{i}\le {X}_{i+,6DOF}$, was calculated. These threshold values are thus to be seen as an upper cutoff of the random distribution. The iterative algorithm consecutively decreased the (absolute) threshold values, recalculated the pass rate until finally reaching a pass rate of $r\ge 95\%.$ This procedure yielded one of an infinite number of possible solutions. For clinical application, however, it is desirable to use a set of thresholds values that limits position deviations as little as possible. The algorithm should therefore numerically select from the manifold of possible solutions (solutions satisfying $r\ge 95\%$) one set that is optimized with respect to its magnitude. We chose to do this optimization in two ways: firstly, by applying a local gradient criterion (steepest descent method), and secondly, by repeating the iteration with randomly generated start values and step sizes.

#### Steepest descent

The gradient based approach was similar to a steepest descent algorithm that determined the threshold decrease of every DOF (positive and negative) ${X}_{i\pm }$ at every iteration step n according to a weight factor ${w}_{{X}_{i\pm }}$. The weight factor was proportional to the slope of the DOF with respect to the pass rate change ${\Delta }\text{r}/{\Delta }{X}_{i\pm }$ in relation to the average pass rate change of all DOFs. In this way, DOFs that were more sensible to position deviations were preferably decreased, resulting in a total decrease of thresholds that was as little as possible. For proper calculation of the mentioned weight factor, translational and rotational DOFs were put into a relation by means of normalization. Shift values ${S}_{i}$ and threshold values ${X}_{i\pm }$ were normalized by the mean values of the results of the 1DOF analysis (where means were calculated separately for translational and rotational DOFs):

$$\begin{aligned} &{X}_{i\pm ,6DOF} \to \frac{{X}_{i\pm ,6DOF}}{{\stackrel{-}{X}}_{i,1DOF}}{\stackrel{-}{X}}_{i,1DOF} \\ & \quad   =  \left\{\begin{array}{c}\frac{1}{6}\sum _{\pm }\sum _{k=1}^{3}\left|{X}_{k\pm ,1DOF}\right|,\: 1\le i\le 3\\ \frac{1}{6}\sum _{\pm }\sum _{k=4}^{6}\left|{X}_{k\pm ,1DOF}\right|,\: 4\le i\le 6\end{array}\right.\end{aligned}$$

The decrease rate was determined by the step size ${\Delta }{X}_{i}$ and the weight factor ${w}_{{x}_{i\pm }}$. For every iteration cycle, the new (dimensionless) threshold values were thus determined by

$$\begin{array}{c}{X}_{i\pm ,6DOF}\left(0\right)= {X}_{i\pm ,6DOF,0}\\ { X}_{i\pm ,6DOF}\left(n+1\right)={X}_{i\pm ,6DOF}\left(n\right)-\varDelta {X}_{i\pm }{w}_{{x}_{i\pm }} \end{array}$$

$${ w}_{{x}_{i}\pm }=\pm \left|\frac{{\Delta }\text{r}}{{\Delta }{X}_{i\pm }}\right|{\left(\frac{1}{12}\sum _{\pm }\sum _{j=1}^{6}\left|\frac{{\Delta }\text{r}}{{\Delta }{X}_{j\pm }}\right|\right)}^{-1}$$

where $\sum _{\pm }$is a sum over positive and negative threshold values.

#### **Random** parameters and normalized magnitude

This steepest descent procedure is somehow similar to locally optimizing the magnitude of the selected set of thresholds with respect to a metric defined by

$$\parallel {\overrightarrow{X}}_{6DOF} \parallel =\sum _{\pm }\sum _{i=1}^{6}\frac{\left|{X}_{i\pm }\right|}{{\stackrel{-}{X}}_{i,1DOF}}$$

For further optimization, we repeated the above mentioned procedure for 10,000 runs with randomly generated start values (with$\left|{X}_{i\pm ,1DOF}\right|\le \left|{X}_{i\pm ,6DOF,0}\right|\le$ 5 mm/5°) and step sizes ($0.01\le {\Delta }{X}_{i}\le 0.2$, dimensionless units) and chose the solution with the highest normalized magnitude $\parallel {\overrightarrow{X}}_{6DOF} \parallel$. It is thus a way of approximating the set of threshold values (that satisfy $r\ge 95\%$) with the greatest magnitude with respect to the above metric. This analysis was repeated for subgroups of the patient collective, categorized by the side of the treatment (left or right) and whether lymph nodes were irradiated or not.

### Data analysis

The two-sample student t-test for unequal variances (Welch’s t-test) was applied to the data in order to check for statistical significance in the asymmetries for positive and negative shifts. Results were rated as not significant (p ≥ 0.05) and significant (p < 0.05). Data analysis and plotting were performed with Anaconda 3.1/Python 3.4.

## Results

### 1DOF analysis

**Fig. 2:**
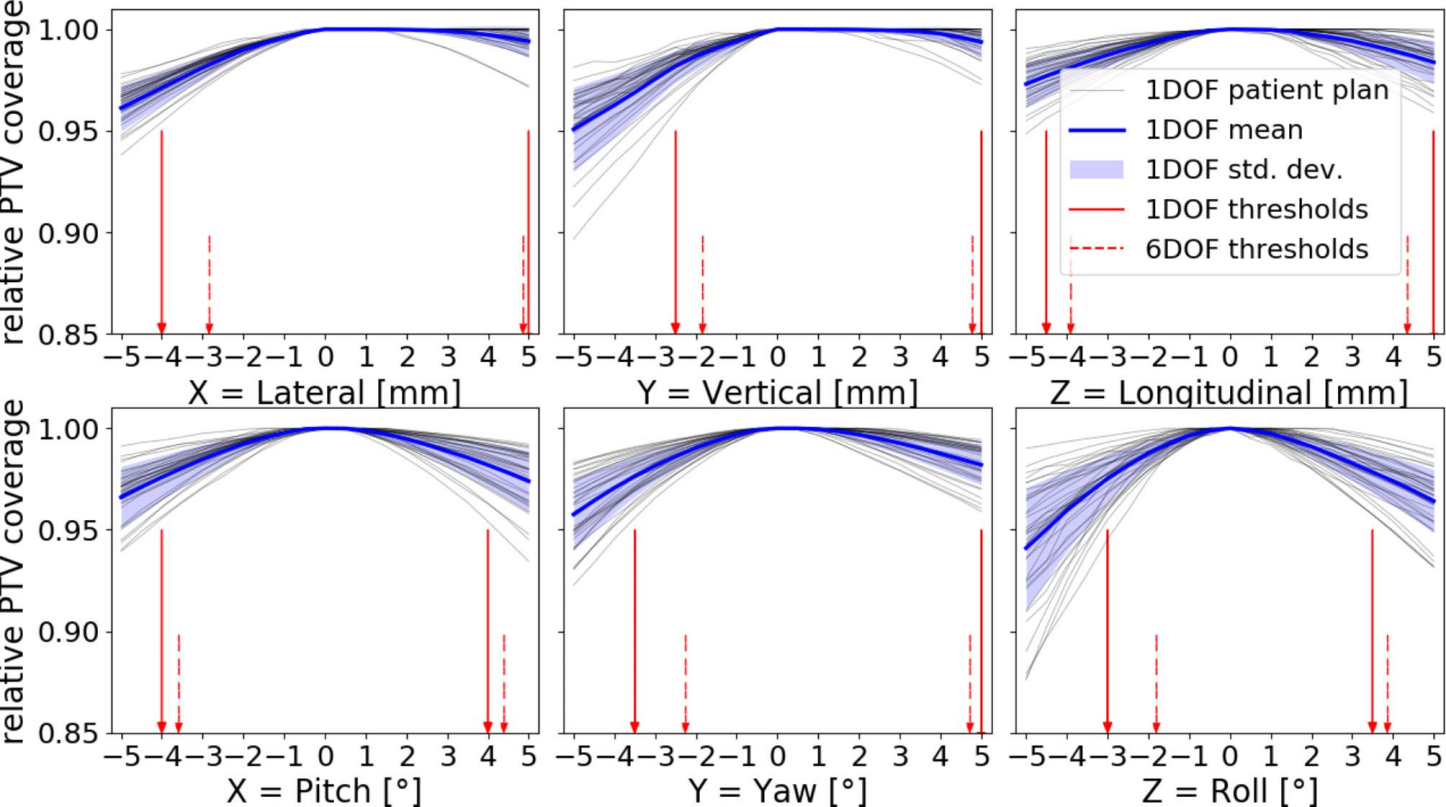
Relative PTV dose coverage for varying patient misalignment for left-sided tumor locality. Individual patient plans, their mean and standard deviation (blue) are marked for the 1DOF case. Shift threshold values, based on dose coverage constraints, marked in red with arrows for the 1DOF and the simultaneous 6DOF case. When threshold values were equivalent to the borders of the evaluated ranges (and thus not a calculated solution), a line instead of an arrow was drawn

Partly drastic changes of relative PTV coverage and OAR dose were observed for the dose distribution resulting from simulated patient motion. In the following, the results of left-sided tumors are presented in detail and a remark is given where the results of right-sided tumors differed qualitatively. For the 1DOF shift analysis, substantial decreases of the coverage were found, especially for vertical translations and rotations in roll. Furthermore, pronounced asymmetries between positive and negative shift values were observed, especially for lateral and vertical translations and for yaw rotations (see Fig. 2 for details). For vertical translations, it had a large effect on the dose coverage when patients were positioned too low. For − 5 mm shift, mean value ± standard deviation was V_95%_ = 95.1 ± 2.1%. At the same time, positioning too high did not have a substantial effect on the coverage (V_95%_ = 99.4 ± 0.7% for + 5 mm shift). Welch’s t-test confirmed statistical significance for the difference in the data of positive and negative shifts for the above mentioned DOFs. Via computation on the basis of the pass rate concept, the mentioned differences between the DOFs translated into threshold values with similar properties. For example, for vertical translations the threshold values were asymmetric, namely + 5 mm and − 2.5 mm, and for longitudinal translations they were similar (+ 5 mm and − 4.5 mm). The values for plans with lymph node irradiation were lower than those without. A detailed summary of the obtained shift values, also for right-sided tumors, is given in Table 1.

**Fig. 3:**
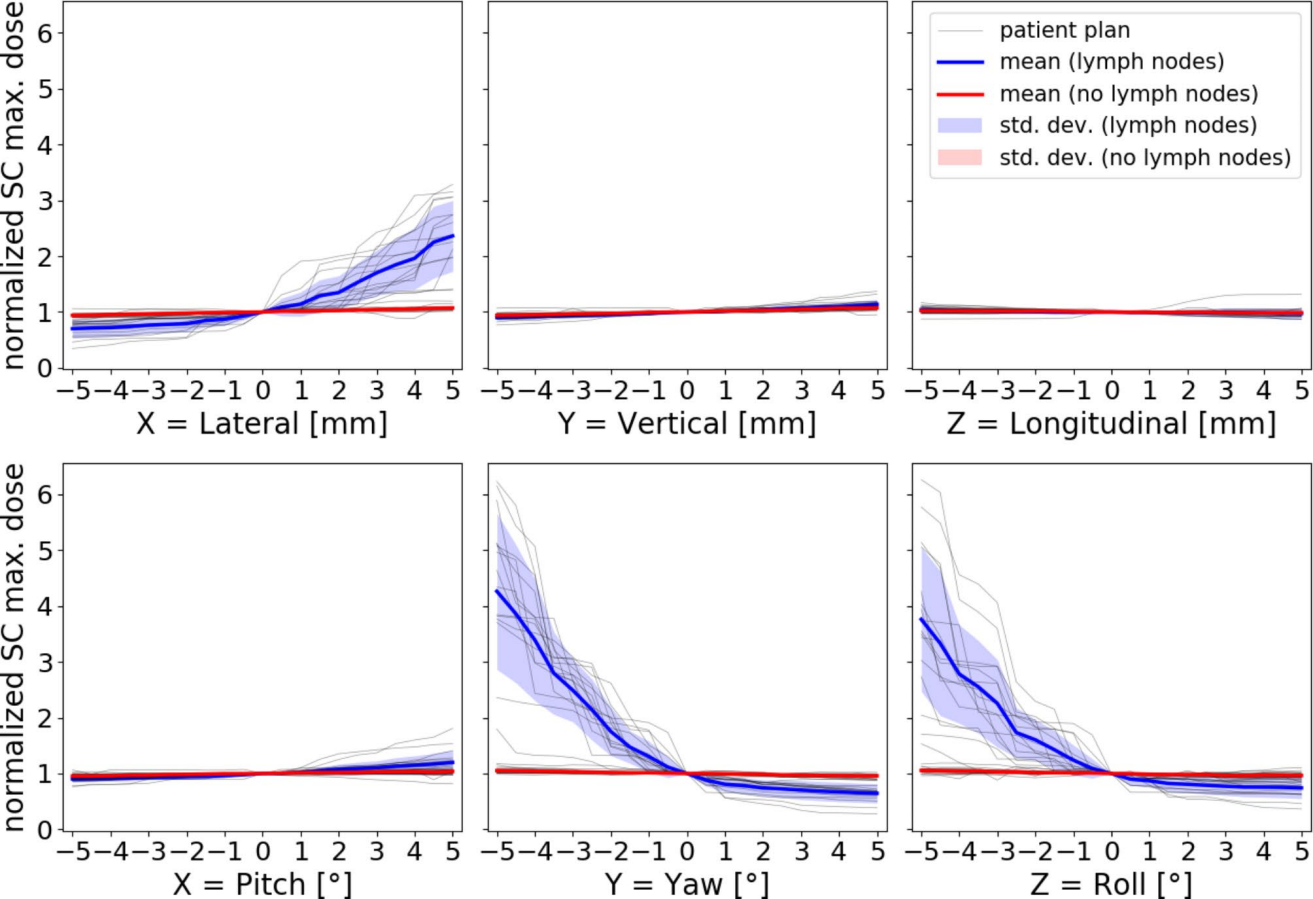
Maximum spinal cord (SC) dose (D_0.01 cm³_), normalized by the value of the original treatment plan, for varying patient misalignment for left-sided tumor locality. The difference between patients with (blue) and without lymph node irradiation (red) is clearly distinguishable as a splitting in two groups for lateral, yaw and roll

Even more pronounced differences occurred when examining the data of the maximum spinal cord dose (D_0.01 cm³_) for patients with irradiated lymph nodes (see Fig. 3). Drastic, asymmetrical increases of the dose were observed for lateral translations and rotations in yaw or roll. For yaw, the cohort mean value was increased by a factor of approximately 4.3 for − 5° relative to the dose of the original treatment plan. The relative maximum value was 6.3 times higher than the original plan, obtained for one patient with a -5° rotation in roll (D_0.01 cm³_ = 27.49 Gy, the planned dose was D_0.01 cm³_ = 4.39 Gy). The dose, however, never exceeded the clinical constraint of 45 Gy. The absolute maximum dose of D_0.01 cm³_ = 35.74 Gy was obtained for a -5° rotation in yaw for a patient whose planned dose was D_0.01 cm³_ = 9.65 Gy. The increase in spinal cord max. dose was negligible for vertical, longitudinal translations and pitch rotations for patients with lymph node irradiation and for all DOFs for patients without lymph node irradiation.

The results for right sided tumor locality were similar to those of the left sided without lymph node irradiation (there were no right-sided tumors with lymph node irradiation in this cohort), but only if certain shifts were inverted, namely lateral translations and rotations in yaw or roll (see Table 1 and additional file 1). Welch t-test confirmed that especially for vertical translations (not inverted) and rotations in roll (p > 0.5 when tested for dissimilarity).

### 6DOF analysis

In order to obtain realistic shift scenarios and threshold values, an analysis of simultaneous 6DOF shifts was performed. The (absolute) threshold values for the 6DOF analysis for left-sided tumors were approx. 0.6 mm and 0.4° smaller on average than the 1DOF values, respectively (see all values, rounded to one decimal, in Table 1). Asymmetries between positive and negative threshold values were preserved or enlarged due to the weighted iteration procedure. For example, for left sided tumor locality, (absolute) threshold values for vertical translations (y) were reduced by 0.2 mm for positive and 0.7 mm for negative shifts, resulting in 6D threshold values of 4.8 mm and − 1.8 mm. Similarity of the values of left and right sided tumor localities (with no lymph node irradiation) under inversion of values for yaw and roll were preserved also for the 6D analysis. The threshold values for plans with lymph node irradiation were lower than for those without (0.61 mm and 0.77° lower an average for the absolute values). This difference was also reflected in differences in normalized magnitude. Similarly, solutions for subgroups allowed for solutions with higher magnitude as the one for the whole cohort (see details in Table 1).

**Table 1:** Maximum shift threshold values for different treatment sites for 1DOF and simultaneous 6DOF shifts, assuring 95% relative target coverage with a pass rate of 95%, optimized with respect to the normalized magnitude for the 6DOF solutions. Additionally, the normalized magnitude of the respective 6DOF solution is given. Translations (x,y,z) given in mm, rotations (P,Y,R) = (pitch, yaw, roll) given in °

	all				left				left, lymph nodes				left, no lymph nodes				right, no lymph nodes			
	1DOF		6DOF		1DOF		6DOF		1DOF		6DOF		1DOF		6DOF		1DOF		6DOF	
x	5.0	-4.5	4.6	-2.8	5.0	-4.0	4.9	-2.8	5.0	-3.0	4.9	-3.2	5.0	-4.5	5.0	-3.2	5.0	-5.0	3.9	-5.0
y	5.0	-3.0	4.7	-1.8	5.0	-2.5	4.8	-1.8	5.0	-2.5	5.0	-0.8	5.0	-3.0	5.0	-2.2	5.0	-3.5	5.0	-1.6
z	5.0	-5.0	4.4	-3.9	5.0	-4.5	4.4	-3.9	5.0	-4.5	3.7	-4.1	5.0	-5.0	5.0	-5.0	5.0	-5.0	5.0	-5.0
P	4.0	-4.0	3.6	-3.5	4.0	-4.0	4.4	-3.6	4.0	-3.0	4.3	-2.7	5.0	-5.0	5.0	-4.1	4.0	-5.0	4.8	-5.0
Y	4.5	-3.5	4.7	-3.0	5.0	-3.5	4.7	-2.3	5.0	-3.5	4.9	-1.6	5.0	-4.0	5.0	-2.8	4.0	-5.0	2.6	-5.0
R	3.5	-3.0	2.2	-1.5	3.5	-3.0	3.9	-1.8	3.5	-3.0	4.1	-1.4	4.5	-2.5	4.8	-1.9	2.5	-4.5	2.4	-4.9
magnitude			9.76				10.83				10.34				11.00				11.30	

## Discussion

The results showed that relevant changes in the dose distribution can occur for relatively small rigid body shifts and that the effects vary strongly between shifts of different DOFs and directions. In particular, the importance of rotational shifts, not thoroughly analyzed in other studies, was demonstrated. The greatest decrease in PTV coverage was observed for vertical translations and rotations in roll. The pronounced asymmetry for vertical translations (and also the slighter asymmetry for lateral translations) is easily understood when taking the planning technique into account. The aperture of the 3D conformal tangential beams used for irradiation was unilaterally extended into the air in vertical and lateral direction (usually 2 cm). Therefore, shifts moving the patient further up (or further left for left sided tumor location) did not move the PTV out of the beams, contrary to shifts moving the patients down (or right). The same holds for the results of the spinal cord dose: here shifts to the left moved the spinal cord into the beams, just as rotations in yaw and roll in negative direction, as can be understood geometrically (see Fig. 1a)). The fact that the asymmetrical thresholds were preserved when inverting lateral, yaw and roll shifts when comparing left and right sided tumor localities, endorse this interpretation.

The results of thresholds values also indicate that treatment plans get less robust with the inclusion of lymph node irradiation and hence require stricter control of the patient’s position. This is especially obvious for rotations (almost 1° smaller threshold values on average, compared to no lymph node irradiation) and stems from the fact that the additional irradiated volume is generally located further away from isocenter. The same holds for the spinal cord max dose if lymph nodes are irradiated, explaining the huge increase for certain rotations (see Fig. 1c)).

The 1DOF threshold values give already a lot of insight into the robustness properties of the treatment plans, especially the differences between and asymmetries in the DOFs. Still strictly 1DOF shifts do not occur in reality, but always in combination with shifts in other DOFs. The simultaneous 6DOF threshold calculation provides a set of threshold values for every DOF that assures that PTV coverage stays above a specified level with a specified probability. As to be expected, the absolute 6DOF values were generally smaller than the respective 1DOF value, as simultaneous shifts require stricter limits on the individual DOF. In some cases, however, the opposite was the case. This is due to the fact that shifts in different DOFs may under certain circumstances effectively compensate.

Aside from that, it has to be underlined that the chosen set is not unique, not even for the random data generated for this study. Its values are dependent on the iterative algorithm that is used. We chose an algorithm that was supposed to select a solution, out of the unlimited number of possible solutions, that allowed for as much as position deviation as possible, because less restrictive limits are desirable in clinical practice. The measure that we used to quantify this was a normalized sum over all threshold values defined by the metric given in the methods section. This metric with a linear sum is better suited for that than one similar to Euclidian distance, since what counts for clinical setup is the range of permitted values. A metric including quadratic values would lead to distorted results.

An approximation of the solution with a maximum of the defined magnitude could be found by randomly generating threshold values and calculating their magnitude. This method is, however, very slow because of the high number of degrees of freedom (since positive and negative values are independent, it is practically 12 DOFs). For one million runs, the best solution yielded a magnitude of 9.44, while mean ± standard deviation was 5.89 ± 0.96. Yet the iterative, steepest descent algorithm is a very fast method to obtain a solution with a comparably high magnitude. The solution is, however, object to numerical features and might converge to a local (rather than the global) maximum. In order to reduce this and to further optimize the solution, we used a steepest descent method with a random component by calculating a large number of runs with randomly generated step sizes and start values. For 10,000 runs, we obtained a solution that converged very quickly to a solution whose magnitude of 9.76 lay much higher than the mean and maximum of the randomly calculated thresholds values. The 6DOF solutions given are thus the thresholds with the maximum magnitude of the solutions that we have calculated, and have a magnitude that is close to a solution with a supposed global maximum magnitude. Other few solutions (less than one out of a million) with similar or higher magnitude exist though, and they might have substantially differing threshold values. Two solutions with slightly differing pass rate or magnitude, respectively, may have significantly differing thresholds values, and one magnitude (or pass rate) is not uniquely defined by one set of threshold values. This is why a characterization of the set of threshold solutions with methods based on mean value and standard deviation, as a method of error propagation in measurements as described in [21], is not meaningful in our assessment.

The random sampling of the shift values for dose calculation was also a way of coping with the large number of degrees of freedom in the simultaneous 6DOF case. It would be computationally too cost intensive if the 6DOF shifts were calculated in a similar way as for the 1DOF analysis.

Our approach is, in terms of the summary of the 2019 ESTRO working group on the matter, a probabilistic *a posteriori* robustness evaluation using a retrospective analysis of plans for different treatment sites [8]. Similar to this study, big differences between planned and administered dose were found. The mentioned lack of benchmark agreement on necessary safety levels [8, 9] make it generally hard to compare our results to other studies. Jensen et al. found that robustly optimized VMAT plans were more robust than 3D conformal plans. Yet they analyzed the robustness against observed shifts for DIBH patients during breath hold, which are usually smaller than for free breathing [12]. Similarly, Mizuno et al. found that automated breast planning plans were more robust in terms of target coverage and homogeneity than forward planned field in field plans, but only for shifts up to 5 mm [13]. McGowan suggested an error-bar-dose-distribution approach for incorporation of robustness in plan evaluation with a database of site-specific criteria [10]. Patient motion during breath hold was also examined by SGRT and dosimetrically evaluated by Tang et al., to our knowledge the only one that included rotations in the analysis [16]. They found large decreases in dose coverage for the internal mammary lymph nodes, thus at the edge of the target volume. There are similar studies for stereotactic treatment of the lung [18] and the brain [17]; both found average coverage decreases of approximately 10% for the observed shifts. Kügele et al. investigated isocenter shifts during breath hold and analyzed the dosimetric effect of certain percentiles of the observed shifts on target volume and OAR dose [15]. They observed shifts above 5 mm and substantial coverage decreases and increased heart dose. Other studies have used an approach of sampling uncertainties from a probability distribution in order to evaluate the impact on the dose coverage statistically [9, 14]. They used a truncated normal distribution though. In this way, the (assumed) probability of the occurrence of different shifts is incorporated in the analysis. This is to some respect different to our approach used in order to find maximum shift values. The actual probability of the occurrence of the shifts was not simulated, but sampled homogenously in order to obtain statistically representative data also for larger shifts. It is not obvious how to choose the probability distribution for the shifts, therefore a uniform distribution as a kind of worst-case approximation was used.

Our study is the first to systematically evaluate the treatment plan robustness with respect to differences between the DOFs, its symmetry properties and tumor locality dependence, as well as dependence on target volume (with or without lymph nodes). Similarly, rotational shifts had not been thoroughly investigated; our results indicate that they cannot be neglected in a robustness study. The only reporting related to maximum shifts values is found in the study of Kügele et al., where a maximum magnitude of 3 mm was found to decrease heart dose substantially [15]. Notwithstanding, our study is the only to systematically identify threshold values of allowed 6DOF shifts, suitable for application in patient position monitoring as undertaken with SGRT. The threshold values can be used in order to improve the accuracy of the treatment and even allow for the differentiation of subgroups based on tumor locality and target volume.

Although OAR dose was not directly considered in the threshold calculation in this study, the analysis could be extended in order to include them. Figures illustrating the dependence of heart, lung and contra-lateral mamma are attached in the additional files (2, 3, 4). We have limited ourselves here to the spinal cord for matters of simplicity for illustration of our approach. For the spinal cord dose, there is a very clear dose constraint set by clinicians in our institution. This limit was checked for the threshold values calculated from the dose coverage constraints. Since it was never exceeded, the spinal cord dose deviations are effectively included in the threshold determination. Nonetheless, the approach may easily be amended for the inclusion of other constraints (like on the dose to the coronary arteries, which were not delineated in our institution), but these constraints are subject to discussion as well. Similarly, going beyond this study, an analysis of the shifts as observed by SGRT would be of interest. Our proposed procedure could be used individually for every treatment plan in order to access its robustness and identify those that would require threshold values that are clinically not feasible.

A limit of this study is the use of rigid deformations in order to simulate dose deviations. Deformations, as caused by incorrect arm positioning for example [22], however, may have a big impact on the dose distribution. Nonetheless, the objective of this study was to obtain a better understanding of the influence of rigid patient motion in the way we can observe it during treatment with SGRT. The AlignRT’s algorithm is intrinsically non-deformable, so the shifts calculated and used during treatment for position correction are as well based on this non-deformable approximation. Since we wanted to obtain threshold values for SGRT use, we decided to do the dose recalculation with a rigid registration as well. In this approach, deformations may be considered as an additional source of dose deviations, independent of rigid motion, that cannot be analyzed with the given tools. We also chose to use the PTV as reference for the coverage constraints, although the clinically relevant measure is the coverage of the CTV. The patients in this study received whole breast irradiation, for which the PTV is directly delineated in our institution because of the similarity to the CTV in many directions. Nonetheless, we want to underline that the objective of this study was finding a method for coping with position deviations and that our approach is easily adaptable to other constraints, target volumes and clinical necessities.

## Conclusion

The robustness of 3DCRT breast cancer treatment plans and the influence of 6DOF rigid shifts in patient positioning on the dose distribution were analyzed by calculating the original treatment plan on shifted CTs. Substantial decreases in target dose coverage and increases of spinal cord dose were found. Differences in the robustness between the DOFs and asymmetries with respect to positive and negative shifts were identified, allowing for the identification of most sensitive DOFs. The results highlight the importance of differentiating between DOFs, of taking asymmetrical sensitivities into account and of controlling rotational shifts, especially if clavicular lymph nodes are irradiated. Based on dose coverage constraints, a practical approach of determining 6DOF shift limits using random sampling of shifts was introduced. A set of threshold values applicable for SGRT based patient positioning and monitoring was identified for every DOF individually and in dependence of treatment site and locality.

### Electronic supplementary material

Below is the link to the electronic supplementary material.

**Additional file 1**. Relative PTV dose coverage for varying patient misalignment for right-sided tumor locality (no lymph node irradiation). Shift threshold values, based on dose coverage constraints, marked in red.

**Additional file 2**. Mean dose of the contra-lateral mamma for varying patient misalignment for left-sided tumor locality.

**Additional file 3**. Mean heart dose for varying patient misalignment for left-sided tumor locality.

**Additional file 4**. Mean ipsi-lateral lung dose for varying patient misalignment for left-sided tumor locality.

## Data Availability

The datasets used and/or analyzed during the current study are available from the corresponding author on reasonable request.

## List of Abbreviations

DOF: Degrees of freedom
SGRT: Surface guided radiation therapy
CBCT: Cone beam computed tomography
TPS: Treatment planning system
P: Pitch
Y: Yaw
R: Roll
OAR: Organ at risk
SC: Spinal cord

## References

[1] Stanley DN, McConnell KA, Kirby N, Gutiérrez AN, Papanikolaou N, Rasmussen K (2017). Comparison of initial patient setup accuracy between surface imaging and three point localization: a retrospective analysis. J Appl Clin Med Phys.

[2] 2 Freislederer P, Kügele M, Öllers M, Swinnen A, Sauer TO, Bert C, Giantsoudi D, Corradini S, Batista V (2020). Recent advances in Surface guided Radiation Therapy. Radiat Oncol.

[3] Wang H, Xu Z, Grantham K, Zhou Y, Cui T, Zhang Y, Liu B, Wang X, Vergalasova I, Reyhan M, Weiner J, Danish SF, Yue N, Nie K (2021). Performance assessment of two motion management systems for frameless stereotactic radiosurgery. Strahlenther Onkol.

[4] Laaksomaa M, Sarudis S, Rossi M, Lehtonen T, Pehkonen J, Remes J, Luukkanen H, Skyttä T, Kapanen M (2019). AlignRT((R)) and Catalyst in whole-breast radiotherapy with DIBH: is IGRT still needed?. J Appl Clin Med Phys.

[5] Sauer TO, Ott OJ, Lahmer G, Fietkau R, Bert C. Prerequisites for the clinical implementation of a markerless SGRT-only workflow for the treatment of breast cancer patients. Strahlenther Onkol. 2022;1–8. 10.1007/s00066-022-01966-7. 10.1007/s00066-022-01966-7 PMC9839804 35788694

[6] Yock AD, Mohan R, Flampouri S, Bosch W, Taylor PA, Gladstone D (2019). Robustness analysis for external beam radiation therapy treatment plans: describing uncertainty scenarios and reporting their dosimetric consequences. Pract Radiat Oncol.

[7] Van Herk M, Remeijer P, Rasch C, Lebesque JV (2000). The probability of correct target dosage: dose-population histograms for deriving treatment margins in radiotherapy. Int J Radiat Oncol Biol Phys.

[8] Hernandez V, Hansen CR, Widesott L, Bäck A, Canters R, Fusella M (2020). What is plan quality in radiotherapy? The importance of evaluating dose metrics, complexity, and robustness of treatment plans. Radiother Oncol.

[9] Korevaar EW, Habraken SJ, Scandurra D, Kierkels RG, Unipan M, Eenink MG (2019). Practical robustness evaluation in radiotherapy–A photon and proton-proof alternative to PTV-based plan evaluation. Radiother Oncol.

[10] McGowan SE, Albertini F, Thomas SJ, Lomax AJ (2015). Defining robustness protocols: a method to include and evaluate robustness in clinical plans. Phys Med Biol.

[11] Sterpin E, Van den Rivas ST, George B, Lee JA, Souris K (2021). Development of robustness evaluation strategies for enabling statistically consistent reporting. Phys Med Biol.

[12] Jensen CA, Roa AMA, Johansen M, Lund J, Frengen J (2018). Robustness of VMAT and 3DCRT plans toward setup errors in radiation therapy of locally advanced left-sided breast cancer with DIBH. Phys Med.

[13] Mizuno N, Yamauchi R, Kawamori J, Itazawa T, Shimbo M, Nishimura K (2022). Evaluation of robustness in hybrid intensity-modulated radiation therapy plans generated by commercial software for automated breast planning. Sci Rep.

[14] Tilly D, Ahnesjö A (2015). Fast dose algorithm for generation of dose coverage probability for robustness analysis of fractionated radiotherapy. Phys Med Biol.

[15] Kügele M, Edvardsson A, Berg L, Alkner S, Andersson Ljus C, Ceberg S (2018). Dosimetric effects of intrafractional isocenter variation during deep inspiration breath-hold for breast cancer patients using surface‐guided radiotherapy. J Appl Clin Med Phys.

[16] Tang X, Cullip T, Dooley J, Zagar T, Jones E, Chang S (2015). Dosimetric effect due to the motion during deep inspiration breath hold for left-sided breast cancer radiotherapy. J Appl Clin Med Phys.

[17] Foster RD, Moeller BJ, Robinson M, Bright M, Ruiz JL, Hampton CJ, Heinzerling JH (2021). Dosimetric analysis of Intra-Fraction Motion detected by Surface guided Radiation Therapy during Linac Stereotactic Radiosurgery. Int J Radiat Oncol Biol Phys Physics.

[18] Heinzerling JH, Hampton CJ, Robinson M, Bright M, Ruiz JL, Symanowski JT (2019). Surface-guided Radiation Therapy (SGRT) during stereotactic body Radiation Therapy treatments (SBRT) of the lung: dosimetric implications of Intrafraction Motion. Int J Radiat Oncol Biol Phys.

[19] Sauer TO, Ott OJ, Lahmer G, Fietkau R, Bert C (2023). Prerequisites for the clinical implementation of a markerless SGRT-only workflow for the treatment of breast cancer patients. Strahlenther Onkol.

[20] Sauer TO, Ott OJ, Lahmer G, Fietkau R, Bert C (2021). Region of interest optimization for surface guided radiation therapy of breast cancer. J Appl Clin Med Phys.

[21] BIPM IEC, IFCC, ILAC, ISO IUPAC, IUPAP. and OIML. Evaluation of measurement data | Guide to the expression of uncertainty in measurement. Joint Committee for Guides in Metrology, JCGM 100:2008.

[22] Kügele M, Mannerberg A, Nørring Bekke S, Alkner S, Berg L, Mahmood F (2019). Surface guided radiotherapy (SGRT) improves breast cancer patient setup accuracy. J Appl Clin Med Phys.

