# Supplementary figures and images for: Plan robustness analysis for threshold determination of SGRT-based intrafraction motion control in 3DCRT breast cancer radiation therapy
Source: Radiat Oncol. 2023 Sep 22;18:158. doi: 10.1186/s13014-023-02325-1 (PMC10517562; doi:10.1186/s13014-023-02325-1)

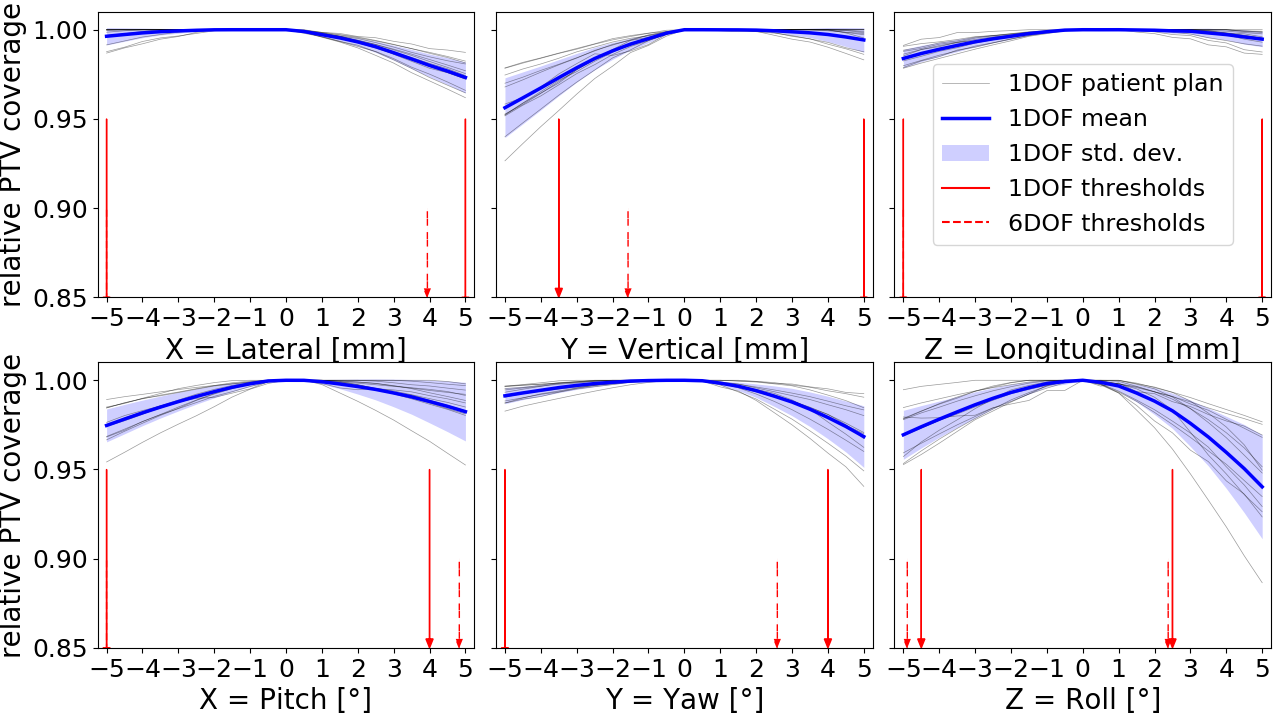

Supplement: Supplementary file 1 — Additional file 1. Relative PTV dose coverage for varying patient misalignment for right-sided tumor locality (no lymph node irradiation). Shift threshold values, based on dose coverage constraints, marked in red. [file 13014_2023_2325_MOESM1_ESM.png]

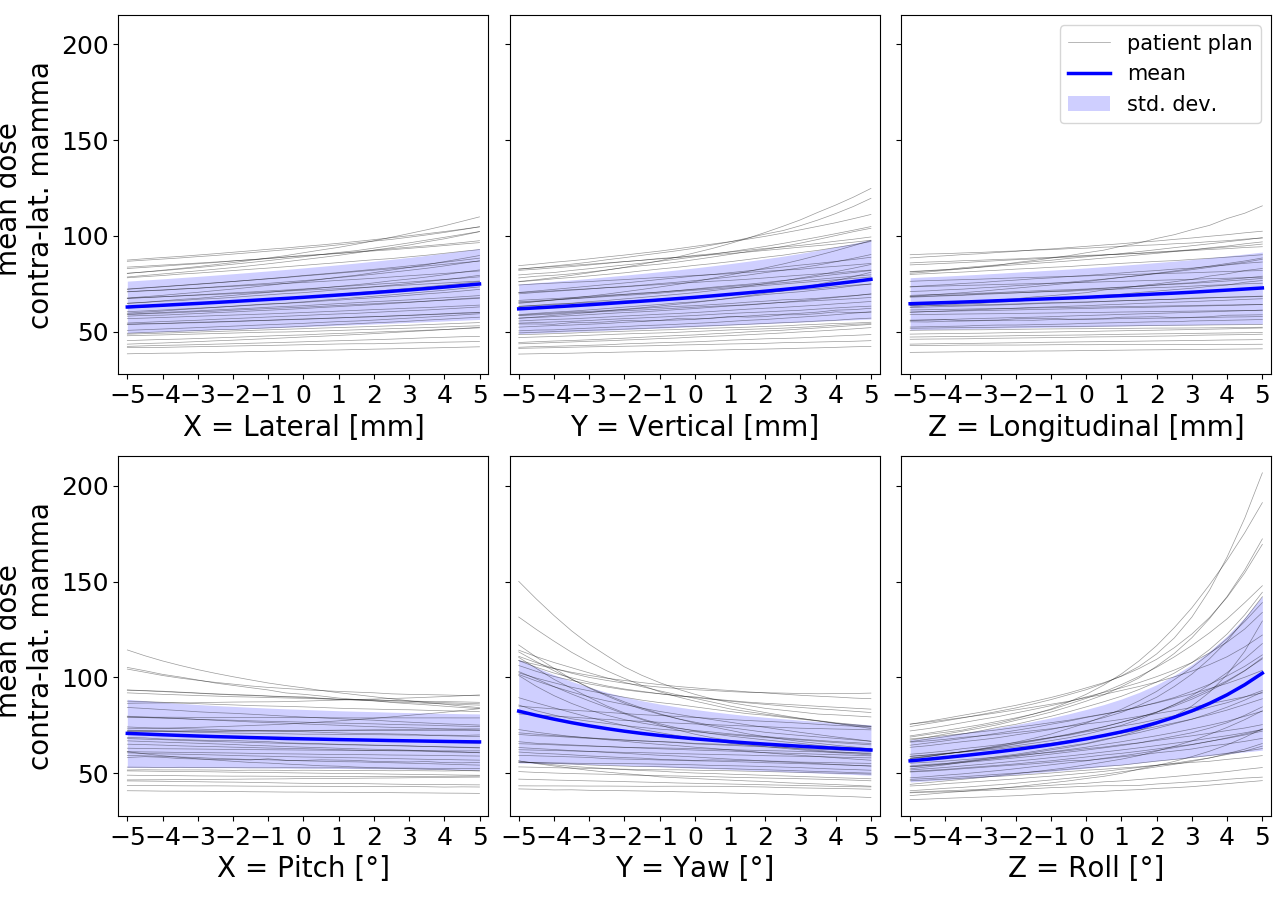

Supplement: Supplementary file 2 — Additional file 2. Mean dose of the contra-lateral mamma for varying patient misalignment for left-sided tumor locality. [file 13014_2023_2325_MOESM2_ESM.png]

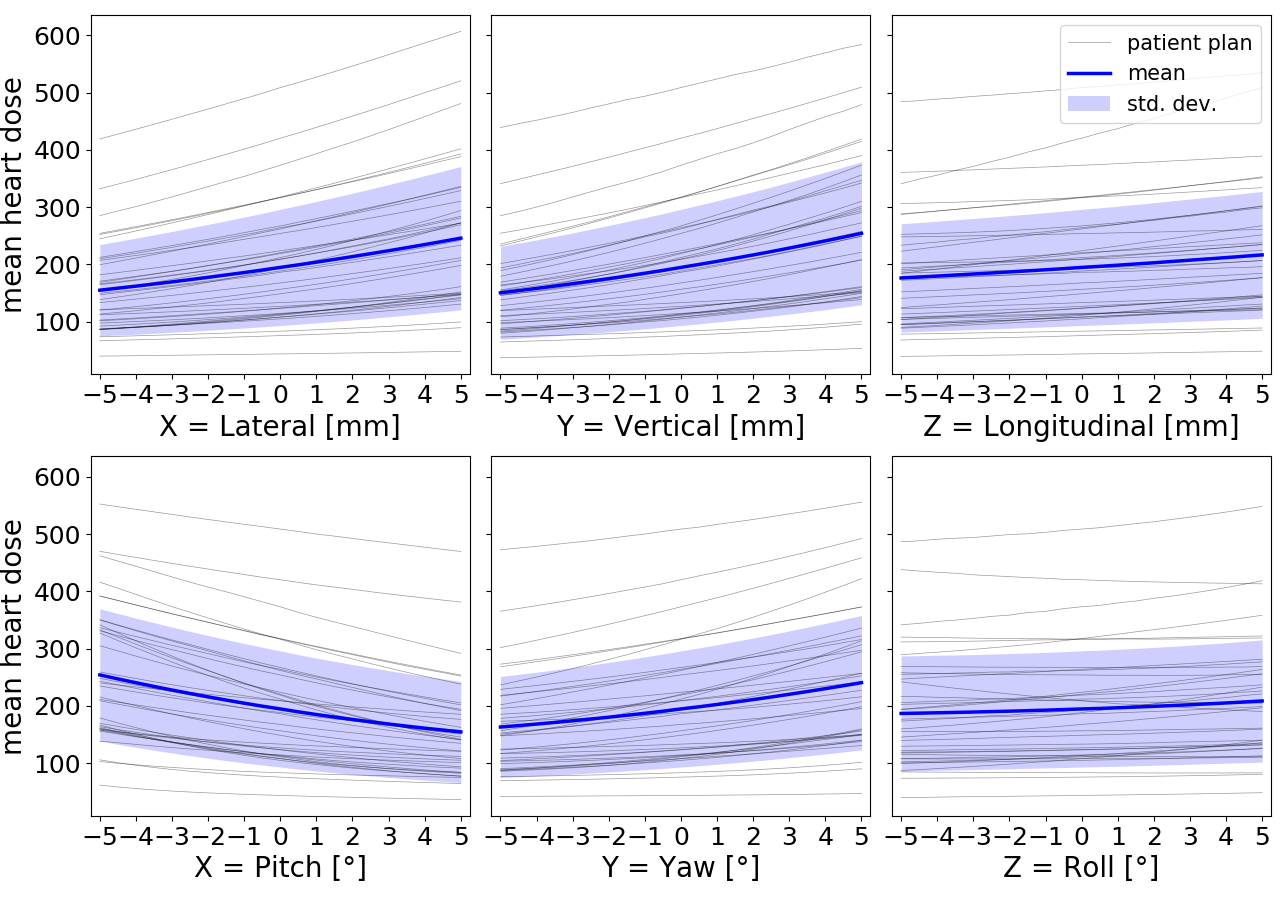

Supplement: Supplementary file 3 — Additional file 3. Mean heart dose for varying patient misalignment for left-sided tumor locality. [file 13014_2023_2325_MOESM3_ESM.png]

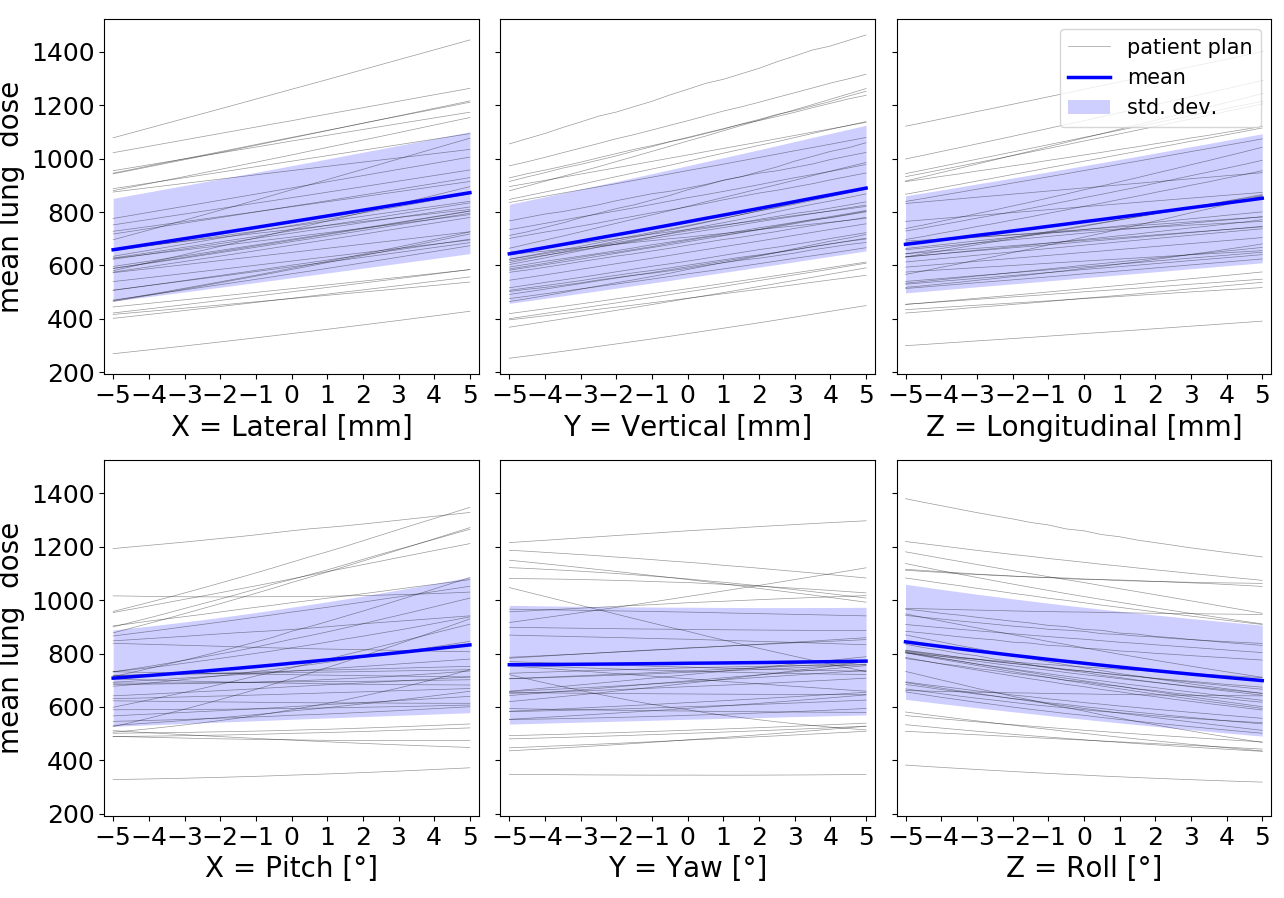

Supplement: Supplementary file 4 — Additional file 4. Mean ipsi-lateral lung dose for varying patient misalignment for left-sided tumor locality. [file 13014_2023_2325_MOESM4_ESM.png]
